# Smad inhibitor induces CSC differentiation for effective chemosensitization in cyclin D1- and TGF-β/Smad-regulated liver cancer stem cell-like cells

**DOI:** 10.18632/oncotarget.16402

**Published:** 2017-03-21

**Authors:** Wei Xia, Chung Mau Lo, Randy Y.C. Poon, Tan To Cheung, Albert C.Y. Chan, Lin Chen, Sitian Yang, George S.W. Tsao, Xiao Qi Wang

**Affiliations:** ^1^ Department of Surgery, The University of Hong Kong, Hong Kong, China; ^2^ Division of Life Science, Center for Cancer Research, and State Key Laboratory of Molecular Neuroscience, The Hong Kong University of Science and Technology, Hong Kong, China; ^3^ School of Biomedical Sciences, The University of Hong Kong, Hong Kong, China; ^4^ State Key Laboratory for Liver Research, The University of Hong Kong, Hong Kong, China

**Keywords:** cyclin D1, Smad2/3/Smad4, cancer stem cells, Smad inhibitor, chemosensitization

## Abstract

Understanding cancer stem cell (CSC) maintenance pathways is critical for the development of CSC-targeting therapy. Here, we investigated the functional role of the cyclin D1-dependent activation of Smad2/3 and Smad4 in hepatocellular carcinoma (HCC) CSCs and in HCC primary tumors. Cyclin D1 sphere-derived xenograft tumor models were employed to evaluate the therapeutic effects of a Smad inhibitor in combination with chemotherapy. Cyclin D1 overexpression confers stemness properties by enhancing single sphere formation, enhancing the CD90+ and EpCAM+ population, increasing stemness gene expression, and increasing chemoresistance. Cyclin D1 interacts with and activates Smad2/3 and Smad4 to result in cyclin D1-Smad2/3-Smad4 signaling-regulated liver CSC self-renewal. The cyclin D1-dependent activation of Smad2/3 and Smad4 is also found in HCC patients and predicts disease progression. A Smad inhibitor impaired cyclin D1-Smad-mediated self-renewal, resulting in the chemosensitization. Thus, pretreatment with a Smad inhibitor followed by chemotherapy not only successfully suppressed tumor growth but also eliminated 57% of the tumors in a cyclin D1 sphere-derived xenograft model. Together, The cyclin D1-mediated activation of Smad2/3 and Smad4 is an important regulatory mechanism in liver CSC self-renewal and stemness. Accordingly, a Smad inhibitor induced CSC differentiation and consequently significant chemosensitization, which could be an effective strategy to target CSCs.

## INTRODUCTION

Cancer stem cells (CSCs) represent a distinct population that can be isolated from tumor tissues and exhibit long-term clonal repopulation and self-renewal capacity [1, 2]. Cancer cells are equipped with apoptotic block, high drug transporter expression, efficient DNA repair and quiescence, which are the mechanisms of therapy resistance. Interestingly, these mechanisms are also active in CSCs [3, 4]. Despite these known mechanisms, the stemness properties of a cancer cell are the core contributors to clinical therapy failure and recurrence. Thus, the therapeutic targeting stem cell maintenance pathways may be an effective means to eliminate CSCs [1], though only if the mechanisms of controlling self-renewal are differentially active in malignant versus normal stem cells [2]. An advanced study has demonstrated that the use of a stemness inhibitor (small molecule) effectively blocked relapse and metastasis in xenografted human cancers [5]. Alternatively, inducing CSC differentiation may be a promising treatment direction. BMP (member of TGF-β family) induces glial differentiation in glioblastomas and consequently attenuates tumor growth [2]. Using all-*trans* retinoic acid (ATRA) to treat BCR–ABL-expressing chronic myeloid leukemia cells induced differentiation to prevent the acquisition of tyrosine kinase inhibitor (TKI) resistance [6]. Thus, targeting cancer stemness and inducing differentiation are novel strategies for cancer therapy.

Cyclin D1 binds and activates CDK4/CDK6 and phosphorylates retinoblastoma protein (Rb) to promote the activation of E2F-responsive genes, which facilitate the transition of cells from the G1 to the S phase of the cell cycle and initiate DNA synthesis. Thus, the primary biological function of cyclin D1 is the promotion of cellular proliferation [7, 8]. Cyclin D1 is also a well-established human oncogene and is important for the development and progression of several cancers [7]. However, this relationship is less apparent in human hepatocellular carcinoma (HCC), for which only CCND1 870G>A polymorphism has been suggested as a risk factor of HCC [9].

Recently, studies have demonstrated the roles of cyclin D1 in stem cell regulation. Specifically, cyclin D1 promotes somatic reprogramming efficiency by enhancing the S phase and cellular proliferation [10, 11], although cyclin D1 does not play a role in promoting induced pluripotent stem cells (iPSCs) maturation towards a complete reprogramming [12]. Constitutive overexspression of cyclin D1 prevents early G1 phase of human embryonic stem cells (hESCs) to initiate differentiation, whereas it induces late G1 phase hESC differentiation by suppressing Smad2/3 transcription and localization [13, 14]. In addition to the correlation between cyclin D1 and Smad2/3 for cell fate coordination via G1 compartmentalization, Smad2/3 is also a key effector in Nodal/Activin signaling, which is an essential pathway for the pluripotency of hESCs [15, 16].

Cyclin D1 is deregulated in many types of cancers, but the functional role of cyclin D1 and its association with TGF-β/Smad signaling in CSC regulation is yet to be defined. In the present study, we demonstrated that cyclin D1-Smad2/3-Smad4 is an important signaling pathway in liver CSC self-renewal. We further evaluated the efficacy of a Smad inhibitor to induce CSC differentiation and chemosensitization to ultimately suppress cyclin D1-expressing sphere-derived xenograft tumors.

## RESULTS

### Cyclin D1 promotes liver CSC spherical cell proliferation and chemoresistance

**Figure 1:**
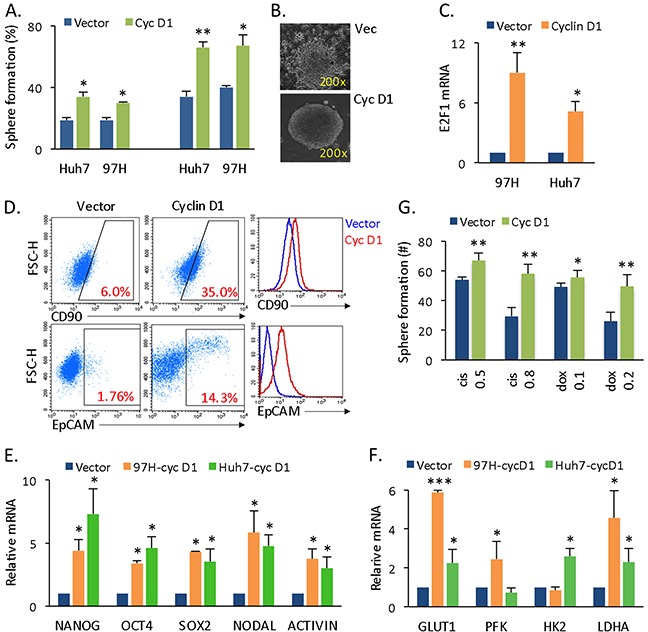
Cyclin D1 expression confers liver cancer cells with CSC and SC features **(A)** Overall sphere formation (left panel) and secondary and third sphere formation from dissected single spherical cells (right panel) in vector- or cyclin D1-expressing Huh7 and 97H cells. **(B)** Phase contrast image of vector- (upper panel) and cyclin D1-expressing (lower panel) liver cancer spherical colonies. **(C)** mRNA levels of E2F1 in vector and cyclin D1-expressing spheres. qRT-PCR data are represented as the mean ± SD, n = 2. **(D)** Flow cytometry analysis of the CD90+ and EpCAM+ population in vector- or cyclin D1-expressing spherical cells shown by both dot blot and histogram. **(E)** mRNA levels of the stemness genes NANOG, OCT4, SOX2, NODAL, and ACTIVIN. qRT-PCR data are represented as the mean ± SD, n = 3 (from different spheres); each experiment was conducted in duplicate. The vector- and cyclin D1-expressing spheres were statistically compared with a paired Student's t test (*, *p* < 0.05; **, *p* < 0.01; ***, *p* < 0.001). **(F)** qRT-PCR of glycolytic genes, GLUT1, PFK1, HK2, and LDHA. **(G)** Sphere formation capability of vector- or cyclin D1-expressing spheres after cisplatin (0.5-0.8 μg) and doxorubicin (0.1-0.2 μg) treatments.

To examine whether cyclin D1 regulates liver CSC proliferation, HCC 97H cells (a HCC tumor-derived primary cell line) and Huh7 cells (Supplementary Figure 1A, 1B) were transfected with lentiviral-cyclin D1 vector. A cancer anchorage-independent spherical colony assay was performed for all experiments because CSCs can be enriched in spherical cells [17]. Cyclin D1 overexpression resulted in increased overall anchorage-independent spherical colony (spheres) formation in two lines of HCC cells (Figure 1A, left panel). The capacity of secondary and third sphere formation from dissected single spherical cells was significantly higher in cyclin D1-expressing than in vector-expressing cells (Figure 1A, right panel), where single sphere formation reflects more self-renewal ability. For the same and longer culturing periods, cyclin D1-expressing spheres maintained a spherical morphology, whereas vector-expressing spheres differentiated (Figure 1B). In addition to promoting self-renewal, cyclin D1 expression promoted spherical cell proliferation, as evidenced by an increased number of cells in the S phase (Supplementary Figure 1C) and increased cell mobility (according to a Transwell assay) (Supplementary Figure 1D, 1E). These phenomenon could be explained by cyclin D1-mediated increased expression of transcription factor E2F1 (Figure 1C), as cyclin D1-CDK4 complex activates Rb and E2F1 to promote cell cycle progression and cellular proliferation [7].

Cyclin D1 expression conferred liver cancer cells with more CSC- and SC-like features, such as increased CD90+ and EpCAM+ liver CSC populations [18] in spherical cancer cells (Figure 1D), as well as in attached cancer cells (Supplementary Figure 1F), and enhanced mRNA levels of the pluripotency-associated genes NANOG, OCT4, SOX2, NODAL and ACTIVIN (Figure 1E, Supplementary Figure 1G). Glycolysis is a metabolic requirement for stem cells [19]. Cyclin D1 also enhanced the expression of glycolytic genes (Figure 1F). More importantly, cyclin D1-expressing spheres were highly resistant to conventional chemotherapeutic drugs (Figure 1G). Taken together, these data show that cyclin D1 expression increased the CSC population and promoted the self-renewal capacity of CSC, which consequently led to chemotherapy resistance.

### Cyclin D1 regulates the activity of Smad2/3 and Smad4

**Figure 2:**
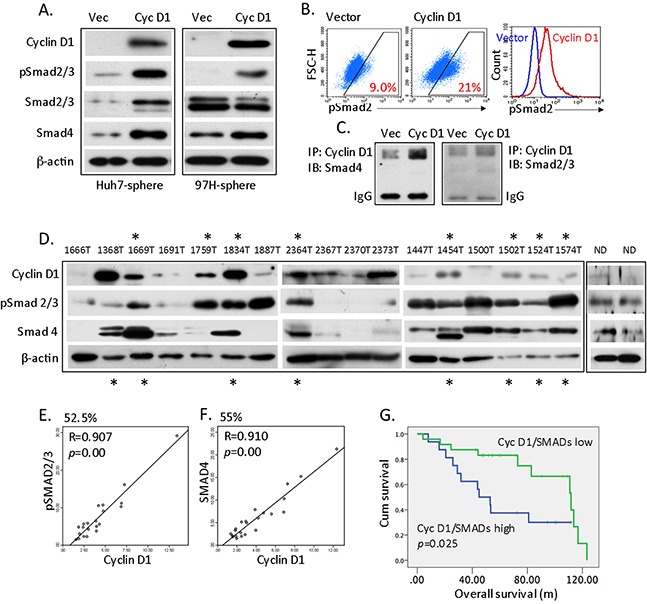
Cyclin D1-dependent activation of Smad2/3 and Smad4 **(A)** Western blot analysis of cyclin D1, phospho-Smad2/3, Smad2/3, Smad4 in cyclin D1 spheres compared with vector spheres. **(B)** Flow cytometry analysis in dot blot and histogram to quantitate pSmad2/3 expression in vector- or cyclin D1-expressing spheres. **(C)** Vector- or cyclin D1-expressing sphere lysates were immunoprecipitated with cyclin D1 antibody; the bound proteins were analyzed using Smad2/3 or Smad4 antibody. **(D)** Representative western blots of cyclin D1, pSmad2/3, Smad4, and β-actin from 40 HCC primary tumor tissues and normal donor (ND) liver tissue. Asterisks indicate an association between the expression pattern of cyclin D1 and pSmad2/3 (top) or Smad4 (Bottom). **(E)** Linear regression analysis of the cyclin D1-dependent expression of pSmad2/3 using quantitated levels of **(D)**. **(F)** Linear regression analysis of the cyclin D1-dependent expression of Smad4 in HCC patients. **(G)** Kaplan-Meier analysis of the overall survival of HCC patients comparing high and low cyclin D1, pSmad2/3, and Smad4 expression.

Activin/Nodel signaling is essential for the self-renewal of hESCs, and Smad2/3 is a main effector of this pathway [15, 16]. The levels of cyclin D proteins have been shown to modulate Smad2/3 activity although it is restricting to the G1 phase of the cell cycle in hESCs [13, 14]. We hypothesize that the regulation of cyclin D1 on Smads might not be limited in G1 phase, we next determined whether and how cyclin D1 regulates Smad2/3 and Smad4 in HCC CSCs. In cyclin D1-overexpressing spherical cancer cells, Smad2/3 phosphorylation and the total level of Smad4 were enhanced, as shown by Western blots and flow cytometry (Figure 2A, 2B). Importantly, a co-IP analysis revealed the interaction of cyclin D1 with both Smad4 and Smad2/3, and cyclin D1 precipitated more Smad4 and Smad2/3 proteins in cyclin D1-overexpressing cells than in controls (Figure 2C). These results suggest that (a) cyclin D1 indeed interacted with Smad2/3 and Smad4 *in vitro*; (b) cyclin D1 expression enhanced Smad2/3 phosphorylation and Smad4 expression in liver cancer cells; (c) cyclin D1 may mediate liver CSC features via TGF-β/Smad signaling, a critical pathway for self-renewal. Moreover, cyclin D1 overexpression also led to an increased phosphorylation of Akt but not Erk1/2 (Supplementary Figure 2A, 2B).

### Association between cyclin D1 and Smad2/3 and Smad4 in HCC patients

We next assessed the interaction of cyclin D1 and Smad in HCC primary tumors. To this end, we measured the protein levels of cyclin D1, phospho-Smad2/3, and Smad4 in 40 of HCC tumor tissues. Many HCC patients expressed high levels of the three tested proteins; specifically, high cyclin D1 was related to high pSmad2/3 expression (Figure 2D, asterisk on the top), high cyclin D1 was related to high Smad4 expression (Figure 2D, asterisk at the bottom), and high cyclin D1 expression was related to both high pSmad2/3 and Smad4 expression (Figure 2D, patients 1669T, 1834T, 2364T, 1454T, 1502T, 1524T, and 1574T). Protein levels of cyclin D1, pSmad2/3, and Smad4 of normal liver tissues were also presented (Figure 2D). Based on a linear regression analysis, 52.5% of HCC patients displayed significant cyclin D1-dependent pSmad2/3 expression (Figure 2E, Supplementary Figure 2C), and 55% of patients exhibited cyclin D1-dependent Smad4 expression (Figure 2F, Supplementary Figure 2D). Clinically, high cyclin D1 levels were significantly associated with late-stage HCC, tumor venous infiltration, and ≥ 2 tumor nodules (Table 1). More importantly, overall survival was significantly reduced in patients expressing high levels of cyclin D1, pSmad2/3, and Smad4 (Figure 2G, *p* = 0.025). Thus, cyclin D1 physically and functionally interacts with pSmad2/3 and Smad4 in both HCC cells and HCC primary tumor tissues, which further implicates in the pathogenesis of the disease, such as a poor prognosis for HCC patients.

**Table 1:** Correlation between clinicopathological parameters and cyclin D1

Parameters	Category	Cases (n=40)	Cyclin D1 level (median)	*P*
Tumor UICC7 stage	I-II	30	12.2	0.004
	III-V	10	23	
Tumor size	≤ 20 mm	14	27.5	0.12
	> 20 mm	13	25.3	
Tumor nodules (no.)	1 nodeul	24	12	0.002
	≥ 2 nodeuls	16	32.5	
Venous infiltration	absent	22	11.8	0.04
	present	18	26	

### Smad4 and Smad2/3 confer liver CSC self-renewal capacity

**Figure 3:**
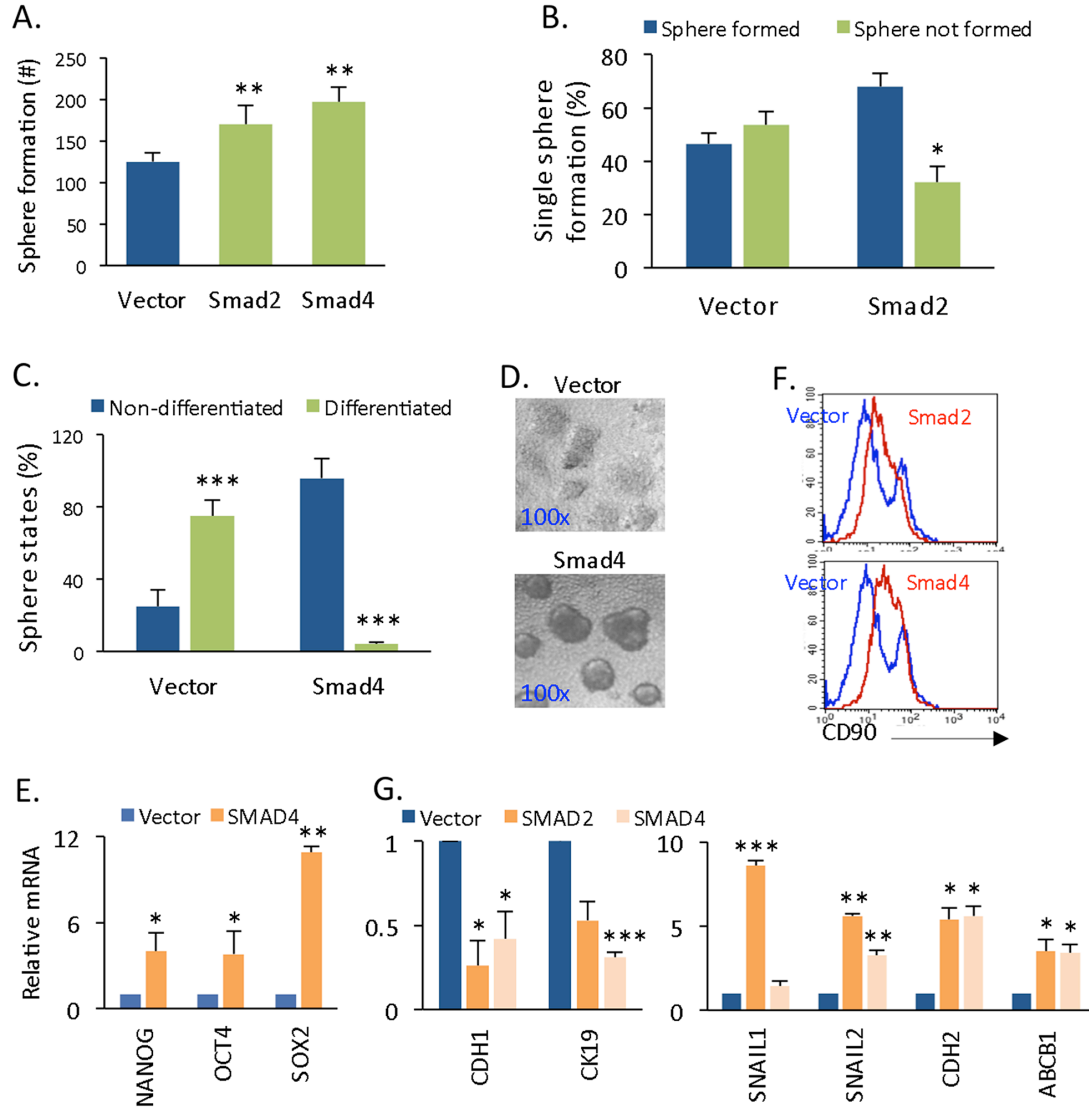
Smad4 and Smad2 expression promotes self-renewal and EMT **(A)** Sphere formation capacity of 97H cells that had been transfected with empty vector and vector encoding Smad4 or Smad2. **(B)** Spheres from **(A)** were dissected for single sphere formation, and the percentages of cells that formed spheres and those that did not were statistically compared. **(C)** Percentages of differentiated versus non-differentiated spheres in vector- or Smad4-expressing HCC cells. **(D)** Phase contrast images of differentiated and non-differentiated sphere colonies. **(E)** mRNA levels of NANGO, OCT4, and SOX2 in vector- versus Smad4-expressing spheres. **(F)** Flow cytometry histogram of CD90+ populations in vector-, Smad2-, or Smad4-expressing spheres. **(G)** mRNA levels of the EMT genes CDH1 and CK19 (left panel); SNAIL1, SNAIL2, and CDH2, and the multidrug resistance gene ABCB1 (right panel) in vector- versus Smad2-expressing and Smad4-expressing spheres.

TGF-β/Smad signaling exerts multiple effects on normal stem cell biology [15], whereas the knowledge of its role in CSCs is limited. Based on the cyclin D1-mediated activation of pSmad2/3 and Smad4 expression (Figure 2), we next assessed whether it is activated Smad2/3 and Smad4 driving the self-renewal of CSC. The overexpression of Smad2 and Smad4 significantly increased the numbers of spherical colonies derived from monolayer cells (Figure 3A). The formed spheres were further dissected into single cells, and 68% of single Smad2-expressing cells grew spherical colonies, whereas 53.5% of vector-expressing single cells were unable to grow spheres (Figure 3B). In the same culturing period, 96% of Smad4-expressing spherical cells maintained non-differentiated states, whereas 75% of control spheres differentiated (Figure 3C, 3D). The activation of Smad2/3 and Smad4 are the intracellular effectors of Nordal/Activin signaling, which regulates the self-renewal of stem cells [15, 16, 20]. To assess the effect of Smad expression on stemness features, we measured the gene expression of NANOG, OCT4, and SOX2, which were significantly increased (Figure 3E). Moreover, the liver CSC CD90+ population was also larger (Figure 3F). The results suggest that Smad2/3 and Smad4 control cancer spherical cell self-renewal by promoting CSC sphere proliferation and preventing CSC sphere differentiation. Because TGF-β signaling is known to trigger the expression of epithelial-mesenchymal transition (EMT)-promoting factors [21], we also assessed the expression of epithelial and mesenchymal markers. Accordingly, we discovered that the overexpression of Smad2 and Smad4 promoted EMT gene expression. Specifically, the epithelial markers E-CADHERIN (CDH1) and CK19 (Figure 3G, left panel) were down-regulated, and the mesenchymal markers SNAIL1, SNAIL2, and N-CADHERIN (CDH2) were up-regulated (Figure 3G, right panel). In addition, the expression of the chemo-resistant gene ABCB1, but not that of ABCG2, was enhanced in Smad-expressing cells (Figure 3G, right panel). Thus, via activating Smad2/3 and Samd4, cyclin D1 regulates the self-renewal ability of liver CSCs by enhancing stemness gene expression, growing the CSC population, and promoting EMT.

### Targeting Smad impairs liver CSC self-renewal

**Figure 4:**
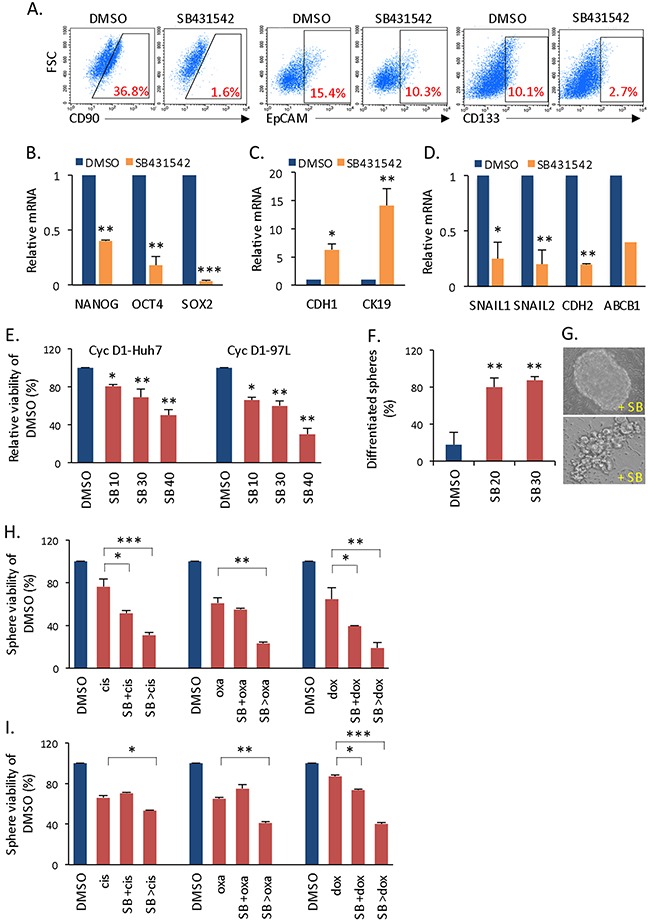
Effects of Smad inhibitor on liver CSCs **(A)** Flow cytometry analysis of the CD90+, EpCAM+, and CD133+ population in cyclin D1-expressing spheres after treatment with the Smad inhibitor SB431542. **(B)** NANOG, OCT4, and SOX2 expression in cyclin D1-expressing spheres treated with SB431542 versus DMSO. **(C)** Expression of the epithelial genes CDH1 and CK19 and **(D)** the mesenchymal genes SNAIL1, SNAIL2, CDH2 and ABCB1 after SB431542 treatment. **(E)** Relative sphere viability of cyclin D1-expressing spheres treated with various doses of SB431542 (10, 30, or 40 μM) versus DMSO. **(F)** Percentage of differentiated spherical colonies after SB431542 (20-30 μM) treatment. **(G)** Phase contrast image of spherical colonies treated with SB431542 versus untreated control. **(H)** Sphere viability of cyclin D1-97H spherical cells treated with cisplatin alone, SB431542 in combination with cisplatin (SB +cis), or SB431542 pre-treatment followed by cisplatin (SB >cis). SB431542 all in low dose (10 μM). **(I)** Same assay as described in (H) in cyclin D1-Huh7 spherical cells.

Given the strong association between cyclin D1 and Smad in controlling liver CSC proliferation, we next applied SB431542 (SB), an inhibitor of activin receptor-like kinase (ALK) receptors, to suppress Smad activation and consequently, CSC self-renewal. The addition of SB431542 to cyclin D1-expressing spheres reduced the populations of CD90+, EpCAM+, and CD133+ cells, which are three liver CSC markers (Figure 4A) (Supplementary Figure 3A); similarly, stemness gene (NANOG, OCT4, and SOX2) expression was also reduced (Figure 4B). Interestingly, Smad inhibitor treatment reversed EMT by increasing E-CADHERIN and CK19 gene expression (Figure 4C), decreasing SNAIL1/2 and N-CADHERIN gene expression (Figure 4D), and functionally reducing cellular mobility (Supplementary Figure 3B, 3C). In addition to promoting cancer metastasis, the induction of EMT has been associated with the acquisition of molecular and functional traits of CSCs [21, 22], such as enriched cancer spherical cells and CSC markers; therefore, EMT reversal at the gene expression level might additionally inhibit CSC proliferation. Furthermore, a high rate of glycolysis is a metabolic signature of CSCs (Warburg effect version 2.0) [23] that is responsible for maintaining the undifferentiated state of these cells [24]. TGF-β/Smad signaling has been known to cross-regulate the PI3K/Akt pathway, which increases glucose uptake and glycolysis [25]. Because cyclin D1 enhanced activation of Akt (Supplementary Figure 2A) and glycolytic gene expression (Figure 1E), we examined the effect of SB431542 on glycolysis. In cyclin D1-expressing spheres, this agent significantly inhibited glycolytic gene expression (Supplementary Figure 3D) and moderately reduced the glycolytic rate, as measured by lactate production (Supplementary Figure 3E). These results all indicate that SB431542 reduced the stemness of cyclin D1-expressing spheres. Consequently, the application of Smad inhibitor significantly but moderately reduced overall CSC sphere formation *in vitro* (Figure 4E). The inhibition was specifically effective in cyclin D1-expressing spheres but not in parental spheres (Supplemenatry Figure 3F). In addition, Mek-Erk inhibitor (U0126) did not inhibit cyclin D1-expressing spheres (Supplementary Figure 3G). These data not only further prove the cyclin D1-mediated activation of Smad2/3 but also demonstrate that cyclin D1-Smad is a functional pathway that regulates liver CSC self-renewal.

### Smad inhibitor induces CSC differentiation and chemosensitization

The observed cyclin D1-Smad-mediated CSC self-renewal provides the logical basis for targeting Smad to inhibit CSC proliferation. However, Smad inhibitor treatment only moderately suppressed the growth of cyclin D1-expressing spheres, and more than 40-60% of cells survived in response to the highest dose of SB431542 (Figure 4E). Furthermore, more than 80% of survived spheres were differentiated cells in response to a low dose of SB431542 (Figure 4F, 4G). Importantly, these surviving spheres lost their stemness characteristics (Figure 4A-4D, Supplementary Figure 3D). Thus, Smad inhibition drives cyclin D1-expressing CSCs into a more differentiated state, letting an expectation that these cells may become sensitization to chemotherapy. To determine whether the addition of Smad inhibitor can chemosensitize highly resistant cyclin D1-expressing spheres (Figure 1F), we first simultaneously applied SB431542 (SB) and cisplatin (cis) (SB +cis). The addition of low-dose SB431542 (10 μM) and cisplatin resulted in a survival rate of over 50%-70% in 97H-cyclin D1 spheres (Figure 4H) and Huh7-cyclin D1 spheres (Figure 4I). We then pre-treated cells with a low dose of SB431542 (10 μM) to induce the differentiation of cyclin D1-expressing spheres, which was followed by chemotherapeutic drug treatment (SB >cis). This strategy significantly sensitized cells to three therapy drugs cisplatin, oxaliplatin (oxa), and doxorubicin (dox) in both 97H- and Huh7-cyclin D1 spheres (Figure 4H, 4I), and the lowest survival rate was less than 20% in 97H-spheres (Figure 4H). When spheres were cultured for longer periods before the addition of SB431542 and subsequent addition of therapy drugs, Smad inhibitor continued to effectively induce chemosensitization (Supplementary Figure 3H). In highly resistant spherical cancer cells, which results from enhanced self-renewal via cyclin D1 and cyclin D1-dependent activation of Smad2/3 and Smad4 (Figures 1, 2, 3), inducing differentiation with Smad inhibitor is a critical tactic for turning resistant CSCs into therapy-sensitive cells. Thus, impaired self-renewal and the induction of differentiation are the molecular basis by which combination therapy eliminates CSCs.

### Differentiation and chemosensitization suppresses cyclin D1-expressing xenograft tumor

**Figure 5:**
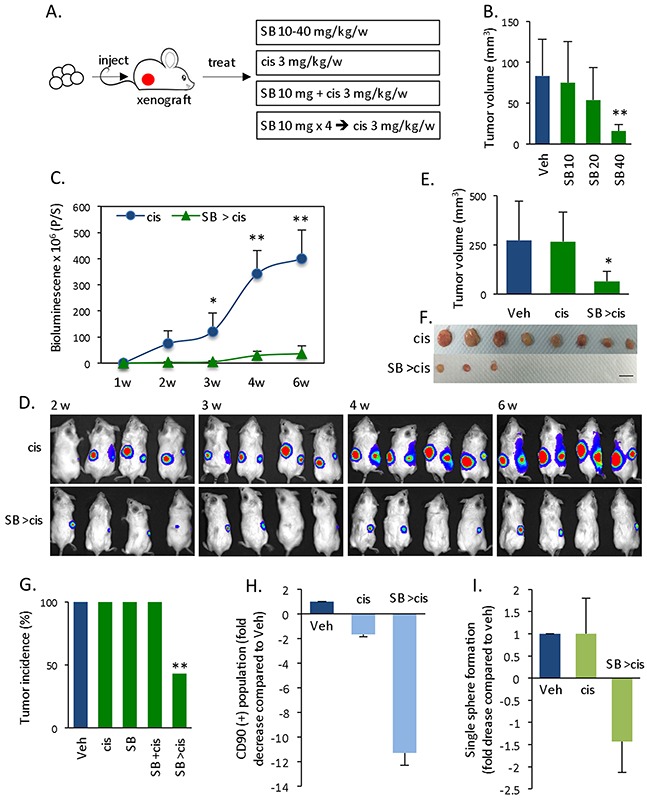
*In vivo* effects of Smad inhibitor-mediated chemosensitization **(A)** Schematic experimental setup for the treatment of a cyclin D1 sphere-derived xenograft tumor model. **(B)** Tumor volume 4 weeks after treatment with different doses of SB431542 alone. **(C)** Tumor growth kinetics based on the luciferin bioluminescence signal in cisplatin versus pre-SB431542 followed by cisplatin treatment. Data are the means ± SD; n = 8 (injection). **(D)** Representative tumor images based on the luciferin bioluminescence of **(C)**. **(E)** Statistical comparison of the tumor volume at the endpoint (6 weeks). **(F)** Representative tumor mass **(C)** at the endpoint. Scale bar = 1 cm. **(G)** Summary of tumor incidence (%) of respective treatment groups: vehicle, cisplatin alone, SB431542 alone, SB431542 + cisplatin simultaneously (SB +cis), or SB431542 pretreatment followed by cisplatin (SB >cis). **(H)** The xenograft tumor tissue was digested at the endpoint to detect the CD90+ population. The percentage of the CD90+ population of the control was defined as 1 for the fold-change of treatment groups. The fold decrease in the CD90+ population in the cis or SB >cis group was calculated as the inverse of the fold change. **(I)** The xenograft tumor tissue was digested and cultured for a few days, followed by single sphere formation. The single sphere formation capacity in the control was defined as 1, the fold decrease in the cis or SB >cis group was calculated as the inverse of the fold change.

A cyclin D1 sphere-derived xenograft tumor model was used to evaluate whether the induction of differentiation may serve as a strategy to target CSCs *in vivo*. Specifically, we compared the effects of therapy in different experimental setups (Figure 5A). When treating with Smad inhibitor alone, only a high dose of SB431542 (40 μg/kg/week) inhibited tumor growth (Figure 5B). Interestingly, administering a low dose of SB431542 and cisplatin simultaneously (SB +cis) did not suppress tumor growth (Supplementary Figure 4A, 4B), and this finding is similar to the results obtained *in vitro* (Figure 4H, 4I). Thus, even combination therapy has little impact when tumor cells are primarily in the CSC state. However, first injecting the same dose of SB431542 and then subsequently administering cisplatin (SB >cis) rendered the combination therapy effective. Moreover, we observed tumor growth kinetics based on the bioluminescence signals of xenograft tumors, which remained low in the SB >cis group, whereas the bioluminescence signals continued to increase in the cisplatin group (Figure 5C, 5D). The tumor volume at the endpoint was also significantly decreased (Figure 5E, 5F). Importantly, we observed a 57% elimination of tumor mass (based on a total of 14 subcutaneous cell injections) in response to Smad inhibitor pre-treatment followed by chemotherapy. The tumor incidence in the SB >cis treatment group was 43%, whereas it was 100% in all other control groups (vehicle, Smad inhibitor alone, cisplatin alone, or SB +cis) (Figure 5G). We hypothesized that Smad inhibitor impaired CSC stemness and induced CSC differentiation to render combination therapy effective. Therefore, we assessed the CSC population and self-renewal ability of xenograft tumor tissue at the endpoint. Compared with control-treated tumor, SB >cis treatment decreased the CSC CD90+ population by 11-fold. In contrast, cis treatment alone resulted in only a 1.6-fold decrease in the CD90+ population (Figure 5H, Supplementary Figure 4C). In addition, overall (Supplementary Figure 4D) and single (Figure 5I) sphere formation was significantly reduced in the SB >cis group, indicating reduced stemness features in this tumor tissue. These results demonstrate that standard chemotherapy is not effective for spherical cancer cell-derived tumors, for which decreasing stemness by inducing differentiation via a SB >cis treatment regimen is the key to chemosensitization.

### *In vitro* Smad inhibition in CSC reduces *in vivo* tumorigenecity

**Figure 6:**
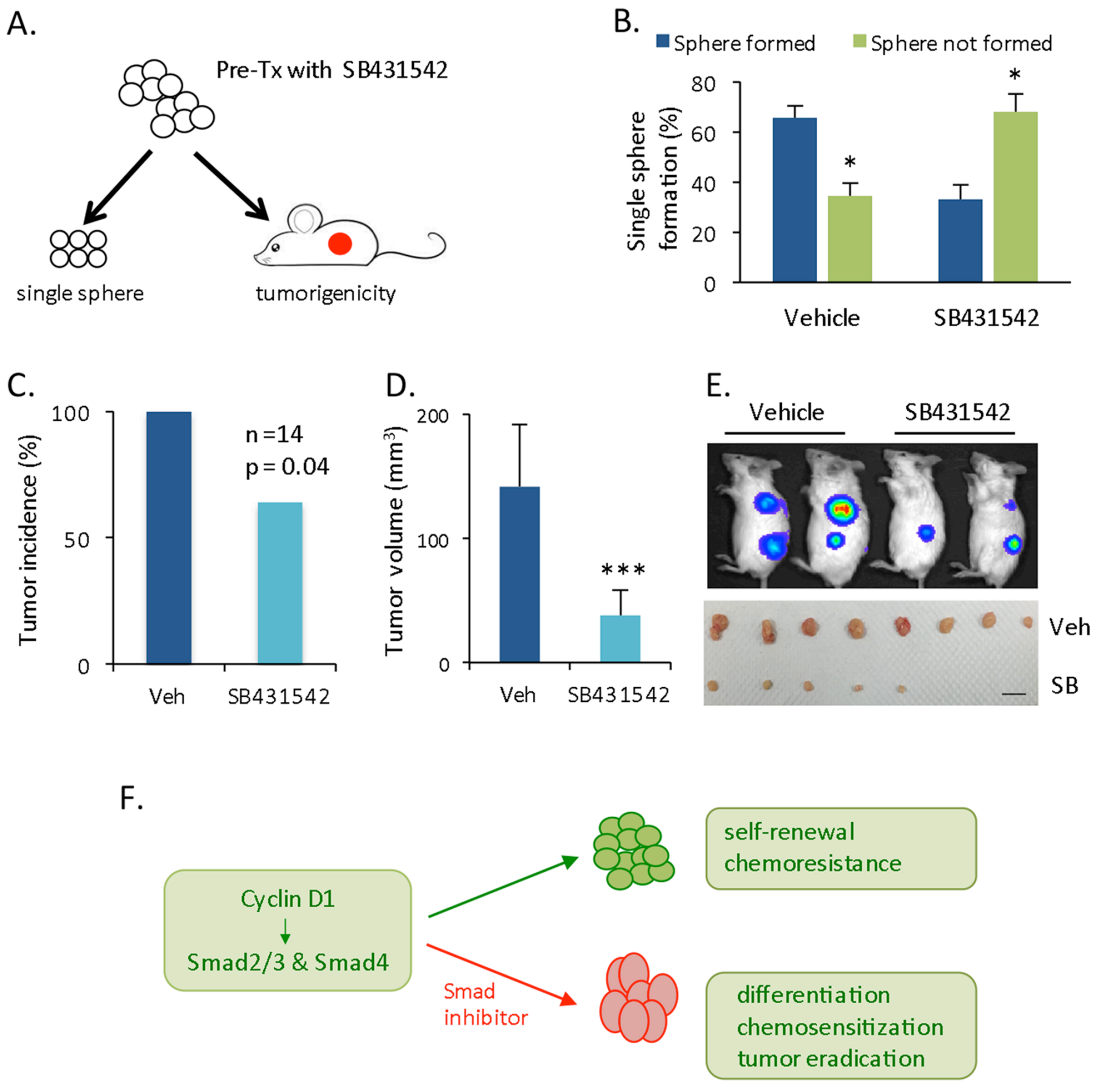
Decreased *in vivo* tumorigenecity of pretreated cells **(A)** Schematic experimental setup. Cyclin D1-spheres were pretreated with SB431542 (20 μM for two times) or DMSO, followed by single sphere formation and *in vivo* tumorigenicity assays, without further treatment. **(B)** Statistical comparison of single cells that formed spheres and those that did not. **(C)** Xenograft tumor incidence (%) at 5 weeks for the injection of pretreated cells (n = 14). **(D)** Statistical comparison of tumor volume at the endpoint. **(E)** Representative tumor size based on bioluminescence imaging (upper panel) and tumor mass (lower panel) at the endpoint. Scale bar = 1 cm. **(F)** Schematic summary of Cyclin D1-Smad2/3-Smad4 axis and therapeutic potential of Smad inhibitor.

For the subsequent investigation, we assessed the *in vivo* tumorigenicity of cells pretreated with Smad inhibitor. Cyclin D1-expressing spheres were exposed to DMSO or SB431542 *in vitro*. All surviving cells were subjected to single cell sphere formation or subcutaneously injected into immunodeficient mice for xenograft tumor formation (Figure 6A). No further *in vitro* and *in vivo* treatment was administered. The single cell sphere formation rate was 66% in the DMSO-pre-treated group versus 33% in the SB431542-pre-treated group (Figure 6B). Xenograft tumorigenicity was determined at 5 weeks. *In vitro* Smad inhibitor pre-treatment partially eradicated tumorigenicity *in vivo* by reducing the tumor incidence, which was 100% in the DMSO group and 64% in the SB431542 group (Figure 6C); Smad inhibitor pre-treatment also significantly reduced tumor volume (Figure 6D, 6E). Thus, Smad inhibitors can drive cyclin D1-Smad-mediated CSC into a more differentiated state that is sensitive to chemotherapy at the cellular level and minimizes tumorigenicity at the *in vivo* xenograft tumor level (Figures 4H, 4I, 5C-5G, 6C-6E).

## DISCUSSION

Cyclin D1, in combination with a great number of oncogenes, CDK inhibitors, oncogenic growth factors, and transcription factors, transforms normal cells into a carcinogenic lineage [26, 27]. New functions of cyclin D1 have been identified, including the enhancement of cellular migration and invasion, angiogenesis, DNA damage repair, and the induction of chromosomal instability [28], which has expanded the known roles of cyclin D1 in cancers. In this study, we demonstrated the biological function of cyclin D1 in liver cancer spherogenesis, which consists of enhancing liver CSC population and the expression of stemness transcription factors, which increases chemotherapy resistance. Thus, in addition to transformation and carcinogenesis, we demonstrate a new role for cyclin D1 in controlling liver CSC self-renewal and proliferation. Cyclin D1-mediated increases in stemness-CSC properties may contribute to therapy failure and cancer recurrence.

TGF-β/Smad signaling is an important effector of a variety of signaling pathways that regulate the self-renewal of normal and cancer stem cells. Cyclin D1 acts as an essential regulator of early cell fate decision during the G1 phase of hESCs by controlling the transcriptional activity of Smad2/3 [13, 14]. Here, we demonstrated that cyclin D1 interacts with and activates Smad2/3 and Smad4 to regulate liver CSC self-renewal. Moreover, this study is the first to demonstrate cyclin D1-dependent pSmad2/3 and Smad4 expression in clinical HCC primary tumors, in which the association implicated a poor prognosis. Therefore, by maintaining stemness and the proliferation of CSCs in HCC tumors, cyclin D1-Smads signaling may be a critical contributor to clinical cancer poor prognosis and conventional treatment failure. Moreover, activated Smad2/3 and Smad4 are functional effectors of several signaling pathways that regulate pluripotency [15, 16, 20]. Similarly, we found that the expression of Smad2/3 and Smad4 directly promotes liver cancer spherogenesis and consequently confers these cells with high stemness and CSC properties. In addition, Smad2/3 and Smad4 expression promoted the EMT phenotype, which further aided the acquisition of self-renewal traits. Thus, liver cancer cells appear to hijack Smad2/3 and Smad4 for CSCs, and this process is closely associated with oncogene cyclin D1 regulation (Figure 6F).

The CSC population has a capacity for long-term repopulation and is highly resistance to chemotherapy or even targeted therapy, which results in therapy failure or cancer recurrence after regression. Therapy approaches that target CSCs have centered on the following: (1) eliminating specific marker-bearing CSCs, (2) treatment with drugs or small molecule that target cancer stemness, such as upstream transcriptional factors or signaling pathways, and (3) the induction of CSC differentiation to attenuate tumor growth [1, 2, 5]. Cyclin D1-dependent activation of Smad2/3 and Smad4 provides the possibility of utilizing Smad inhibitors for targeting CSCs. Indeed, the application of SB431542 reduced cyclin D1-expressing liver CSC spherogenesis and sphere-derived tumor growth, although the inhibition efficacy was limited with no xenograft tumor could be eradicated. However, treatment with a Smad inhibitor significantly impaired the stemness of CSCs, suggesting that the surviving CSCs were differentiated. Based on this hypothesis, the therapy regimen was adjusted by an initial treatment with Smad inhibitor (low dose), followed by conventional chemotherapy. This adjusted treatment resulted in significant chemosensitization. This unique combination therapy regimen not only inhibited tumor growth but it also more effectively eradiated 57% of xenograft tumors. Thus, CSC differentiation may be a critical step in overcoming resistance and ensuring the effectiveness of conventional therapy (Figure 6F). Moreover, the ineffectiveness of the simultaneous administration of low dose Smad inhibitor and cisplatin in both CSC spherogenesis and xenograft models may be associated with the drug administration process, which may not result in CSC differentiation.

In conclusion, this study identified cyclin D1 and cyclin D1-depemdent activation of Smad2/3 and Smad4 regulatory mechamins in HCC spherical cells, which functions in acquisition the CSC characteristics including the self-renewal, CSC markers and stemness gene expression, and chemoresistance. Cyclin D1-dependent activation of Smad2/3 and Smad4 is also reflected in HCC patients with poor prognosis. Targeting Smad followed by conventional therapy induces CSC differentiation resulting in significant chemosensitization in cyclin D1-spheres and cyclin D1-sphere-derived xenograft tumor, highlighting the usage of small molecule (Smad inhibitor) to induce CSC differentiation and chemosensitization could be an effective strategy for targeting CSCs.

## MATERIALS AND METHODS

### Cell culture and CSC sphere assay

The HCC lines MHCC97H (97H) and Huh7 were cultured in DMEM supplemented with 10% FBS (Life Technologies) at 37°C and 5% CO_2_. 97H cells were isolated from a male metastatic HCC patient [29] and transfected with luciferase. Liver cancer spheres were generated in DMEM:F12 (Life Technologies) supplemented with 2% B-27 (Life Technologies), EGF, bFGF (PeproTech), 100 IU/ml penicillin, and 100 μg/ml streptomycin on low attachment or polyHEMA-coated plates for 10-14 days. The resultant cell spheres were dissociated with TrypLE (Life Technologies) and serially diluted for single spherical cell formation. The second generation of single spherical cells was expanded for all further experiments. The number of cells in spherical colonies larger than 50-100 μm was counted. For the chemotherapy drug or inhibitor treatment studies, doxorubicin in 0.2-0.8 μg (Main Luck Pharmaceuticals), cisplatin in 0.25-0.8 μg (Mayne Pharma), oxaliplatin in 1-2 μg (Jiang su heng rui medicine co.), and TGF-β inhibitor SB431542 in 10-40 μM (Merck) were administered 2-3 times. Cell mobility was detected by a transwell migration assay as described previously [30].

### Vectors and transfection

The human cyclin D1 gene was amplified by PCR and cloned into the pWPI-lentiviral vector (Addgene). The lentiviral particles were produced in 293T cells using ViraPower Lentiviral Packaging Mix (Life Technologies) and concentrated by ultracentrifugation (20,000 g). 97H and Huh7 cells were infected with 1 ml of cyclin D1 lentivirus for 24-48 h and then cultured in normal medium. The transfection efficiency was determined based on the GFP-positive populations in both monolayer and spherical cells (Supplementary Figure 1A, 1B). The vectors encoding human Smad2 (pCMV5 Smad2-HA) and Smad4 (pcDNA Flag-Smad4M) were purchased from Addgene. 97H cells were transfected with Smad2 and Smad4 plasmids using Lipofectamine 2000 (Life Technologies) and then subjected to selection with 200 μg of G418 for 4 weeks.

### Western blot and co-immunopricipitation (co-IP)

PVDF membranes containing electrophoretically separated proteins from human HCC tumor tissue and whole cell lysates from spherical cells were probed with antibodies against cyclin D1, phospho-Smad2/3, Smad2/3, Smad4 (Cell Signaling Technology), and β-actin (Sigma-Aldrich). The resultant immune complexes were visualized using enhanced chemiluminescence detection reagents (Bio-Rad). *In vitro* protein interaction was assessed based on co-IP using cyclin D1 and Smad4 or Smad2/3 antibodies. Briefly, 100-200 μg of total cell lysate was incubated with cyclin D1 antibody overnight, followed by incubation with a 50% protein G sepharose bead slurry (Amersham Biosciences) for 2 h. After 5 washes, the immune-complexes were analyzed by immunoblotting with anti-Smad4 or Smad2/3 antibodies.

### qRT-PCR

Total RNA was extracted using an RNeasy kit (Qiagen), treated with DNase I, and then reverse-transcribed with the Transcriptor First Strand cDNA Synthesis Kit (Roche). Quantitative PCR was performed using the Selected SYBR Green master mix (Life Technologies) on an ABI 7900HT Detection System. The PCR primers are listed in supporting information Supplementary Table 1. Gene expression was quantified based on the CT value and normalized to the levels of 18S.

### Flow cytometry

To identify the liver CSC population, the cells were labeled with antibodies against CD90-APC, EpCAM-PE-Cy7 (eBioscience), and CD133 (Miltenyi) and then subjected to a flow cytometry analysis using FACSCalibur (Becton Dickinson). The cell cycle was analyzed using a BrdU and propidium iodide (PI) staining assay after fixation, as previously described [31].

### Clinical HCC specimens

HCC tumor tissue specimens were collected from forty HCC patients diagnosed with stage I-IV pathologic tumor-node-metastasis (TNM) disease [32]. The samples were provided by the Tissue Bank at Department of Surgery at Queen Mary Hospital. The collection and storage of clinical specimens for the Tissue Bank has been approved by the Institutional Review Board of the University of Hong Kong/Hospital Authority of Hong Kong (UW05-3597/I022).

### Xenograft tumor model

Cyclin D1-expressing spherical cancer cells (5 × 10^5^ cells) were subcutaneously injected into 4- or 6-week-old severe combined immunodeficiency (SCID) mice. The mice were randomized to the respective treatment groups. The size of the tumor was monitored based on its luciferin (Gold Biotechnology) signal in an IVIS Spectrum *in vivo* imaging system (PerkinElmer). At the end point, volume and weight of the tumor were measured. Tumor volume was calculated using the formula: tumor volume V = (L × W × W)/2, where L is the length of the tumor and W is the width of the tumor. Mice were peritoneally injected (I.P.) with 10-40 mg/kg SB431542 and 3 mg/kg cisplatin. All mouse experiments were approved by the Committee on the Use of Live Animals of The University of Hong Kong (CULATR 3240-14).

### Statistical analysis

The results for variables are presented as the means ± SD. Treatment groups were compared with controls using a paired or independent Student's t test. The relationship between cyclin D1 and Smad2/3 or Smad4 expression in HCC tumors was analyzed using a linear regression. The correlation of cyclin D1 with clinicopathological parameters was assessed using a crosstab analysis. Differences in patient survival were assessed using a Kaplan-Meier analysis. All analyses were performed using SPSS 21 (SPSS Inc.). A *p* value < 0.05 was considered statistical significance.

## SUPPLEMENTARY FIGURES AND TABLE



## Abbreviation

CSCs: cancer stem cells
HCC: hepatocellular carcinoma
iPSCs: induced pluripotent stem cells
hESCs: human embryonic stem cells
EMT: epithelial-mesenchymal transition
97H: MHCC97H
SB: SB431542
cis: cisplatin
oxa: oxaliplatin
dox: doxorubicin
