# Supplementary material for: Smad inhibitor induces CSC differentiation for effective chemosensitization in cyclin D1- and TGF-β/Smad-regulated liver cancer stem cell-like cells
Source: Oncotarget. 2017 Mar 21;8(24):38811–24. doi: 10.18632/oncotarget.16402 (PMC5503574; doi:10.18632/oncotarget.16402)
Supplement: Supplementary file 1 [file oncotarget-08-38811-s001.pdf]

Smad inhibitor induces CSC differentiation for effective chemosensitization in cyclin D1- and TGF-β/Smad-regulated liver cancer stem cell-like cells

SUPPLEMENTARY MATERIALS

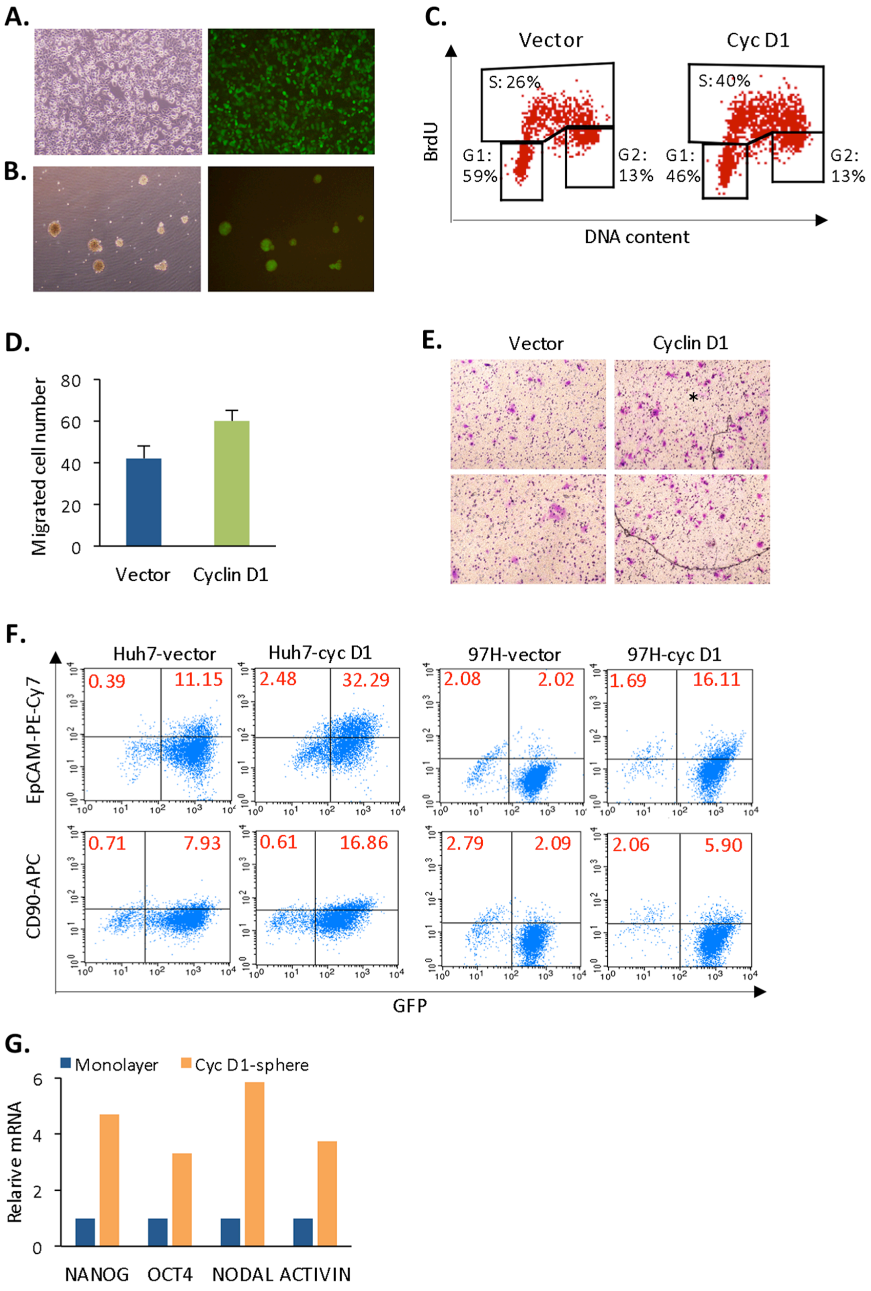

Supplementary Figure 1: Cyclin D1 expression enhances spherical cell proliferation. (A) The cyclin D1 transfection efficiency was high, as indicated by the GFP+ population. **(B)** Cyclin D1-expressing spheres indicated by the GFP+ signal. **(C)** BrdU-labeled S phase population in vector- versus cyclin D1-expressing spheres. **(D)** Cell motility measured using a transwell migration assay in vector- versus cyclin D1-expressing monolayer cells. Data are the mean ± SD, n = 2; each experiment was conducted in duplicate. **(E)** Representative staining of migrated cells. **(F)** CD90+ and EpCAM+ population in vector- versus cyclin D1-expressing Huh7 and 97H monolayer cells. **(G)** mRNA levels of the stemness genes NANOG, OCT4, NODAL and ACTIVIN in monolayer cells versus cyclin D1-expressing spheres.

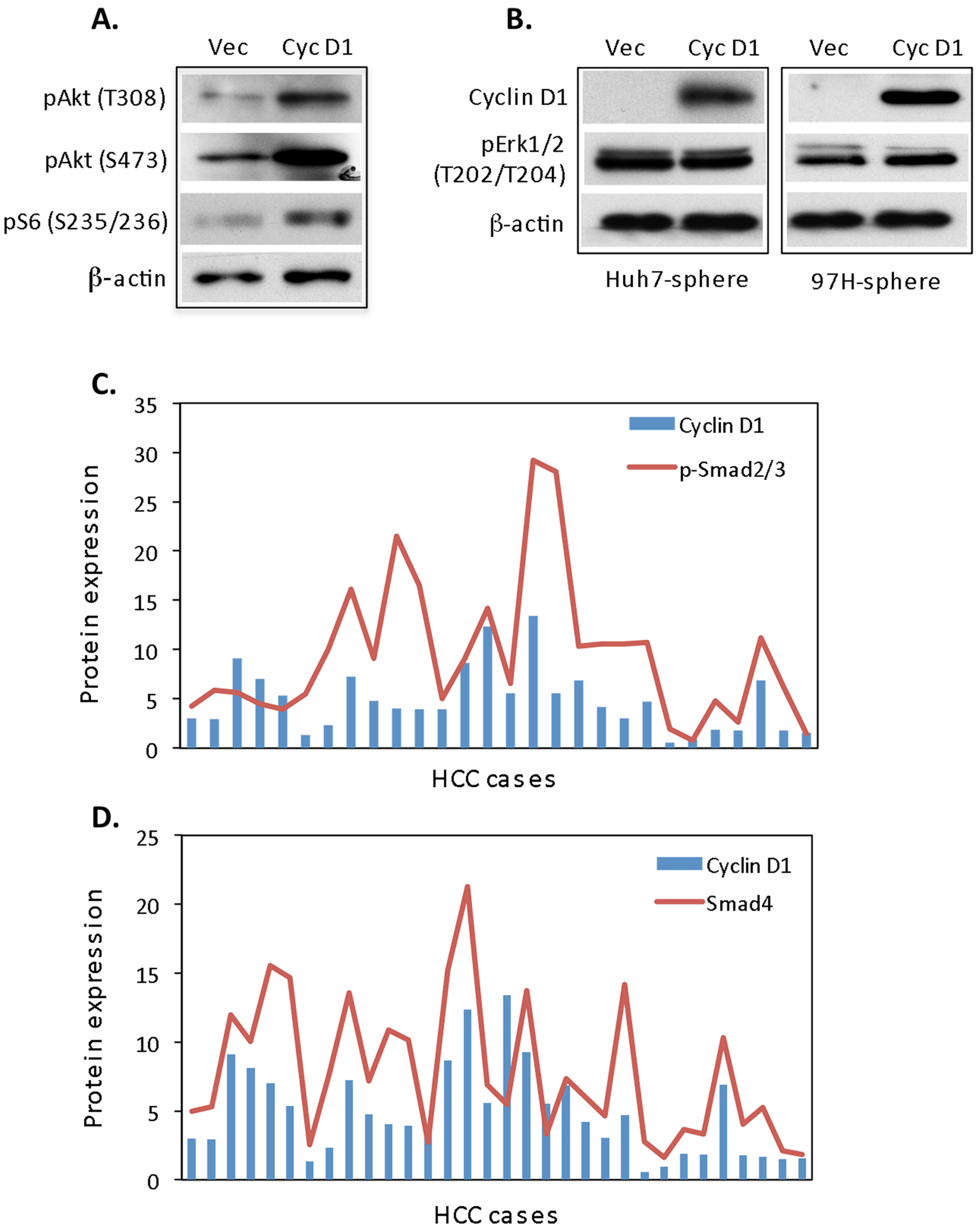

Supplementary Figure 2: Cyclin D1 expression enhanced Akt but not Erk phosphorylation. **(A)** Western blot analyses of pAkt (T308), pAkt (S473), and pS6 (S235/236) in cyclin D1 spheres compared with vector spheres. **(B)** Western blot analysis of pErk1/2 (T202/T204) in vector- and cyclin D1-expressing spheres. **(C)** Representative associated expression pattern of cyclin D1 and pSmad2/3 in HCC primary tumors. **(D)** Representative associated expression pattern of cyclin D1 and Smad4 in HCC primary tumors.

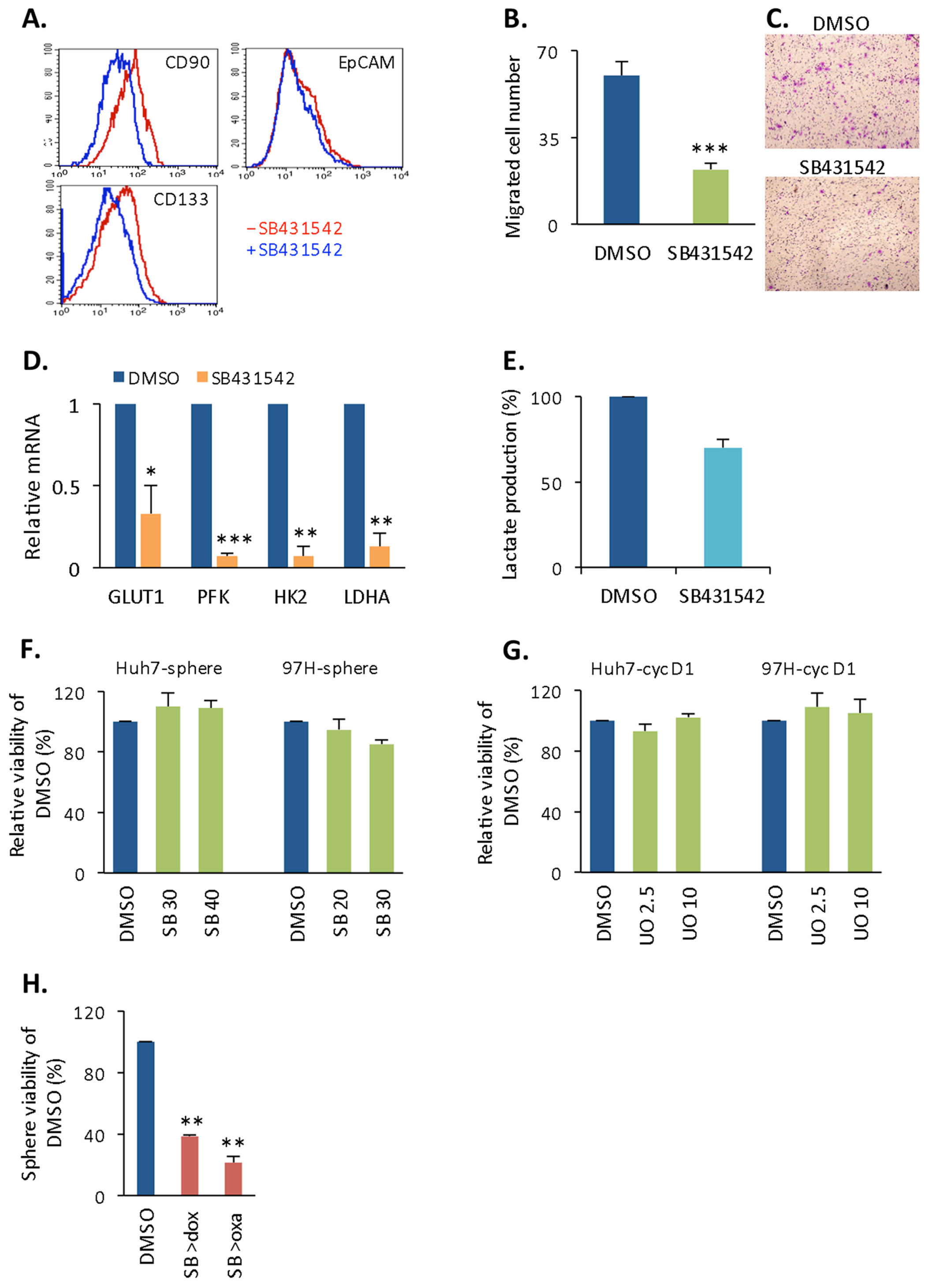

Supplementary Figure 3: Effects of Smad inhibitor. **(A)** Flow cytometry analysis of the CD90+, EpCAM+, and CD133+ population by histogram in cyclin D1-expressing spheres after treatment with the Smad inhibitor SB431542. **(B)** Mobility of cyclin D1-expressing monolayer cells measured using a transwell assay after treatment with SB431542 or DMSO. **(C)** Representative staining of migrated cells. **(D)** qRT-PCR for the glycolytic genes GLUT1, PFK, HK2, and LDHA in cyclin D1-expressing spheres after treatment with SB431542 versus DMSO-treated cells. **(E)** Cyclin D1 spheres were treated with SB431542 or DMSO, and the lactate concentration in the medium was quantitated on day 3. **(F)** Smad inhibitor did not affect the viability of vector spheres after treatment with SB431542 when compared with DMSO-treated cells. **(G)** Sphere viability of cyclin D1 spheres after treatment with the Erk inhibitor UO126 versus DMSO-treated cells. **(H)** Cyclin D1 spheres were seeded and grown for 3 days before SB431542 pre-treatment, which was followed by doxorubicin or oxaliplatin treatment.

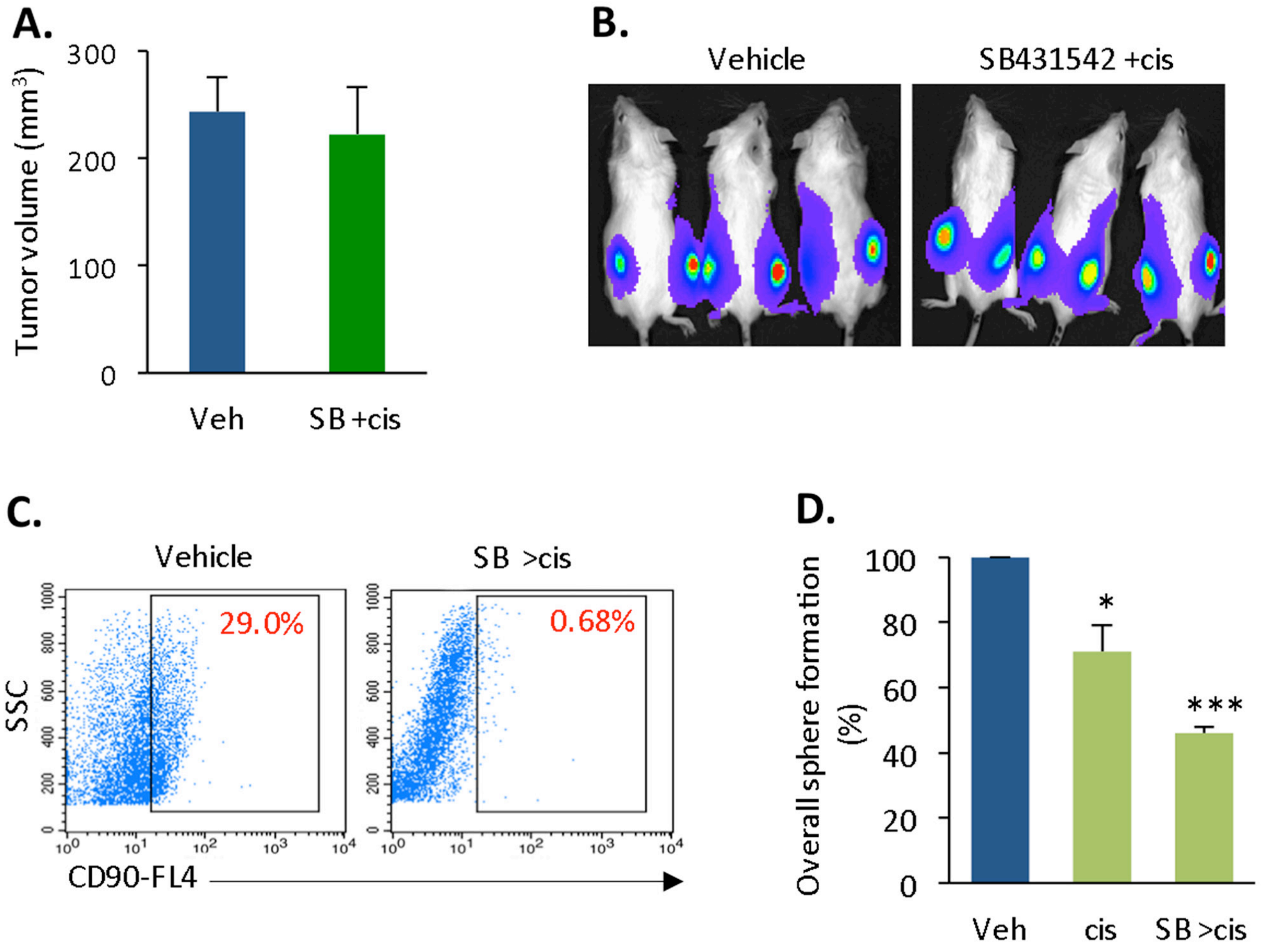

Supplementary Figure 4: Effects of Smad inhibitor in a xenograft tumor model. **(A)** Cyclin D1-derived xenograft tumor volume 4 weeks after 1. vehicle treatment and 2. simultaneous SB431542 and cisplatin treatment. **(B)** Representative luciferin bioluminescence images of **(A)**. **(C)** Representative dot plot of the CD90+ population in cyclin D1-derived xenograft tumors for the vehicle-treated group and the group receiving SB431542 pre-treatment followed by cisplatin treatment. **(D)** The xenograft tumor tissue was digested and cultured for a few days. The overall sphere formation capacity was compared in the vehicle, cisplatin, and SB431542 pre-treatment followed by cisplatin groups.

Supplementary Table 1: Primer sequences

Primer	Sequence (5' to 3')<br>F: forward; R: reverse
CCND1	F: AGCTTCGTTTAAACATGGAACACCAGCTC<br>R: AGCTTGGTTTAAACTCAGATGTCCACGTC
AVTICIN	F: AGACACGGGAGTGCATCTACT<br>R: GCCTATCGTAGCAGTTGAAGTC
NODAL	F: CAGTACAACGCCTATCGCTGT<br>R: TGCATGGTTGGTCGGATGAAA
NANOG	F: CAAAGGCAAACAACCCACTT<br>R: TCTGCTGGAGGCTGAGGTAT
OCT4	F: CTCACCCTGGGGGTTCTATT<br>R: CTCCAGGTTGCCTCTCACTC
SOX2	F: GCTGCGAACAGTCAGACAGA<br>R: ACCTCCCGTCCAAGGTAGG
GLUT1	F: AACTCTTCAGCCAGGGTCCAC<br>R: CACAGTGAAGATGATGAAGAC
PFK1	F: GGTGCCCGTGTCTTCTTTGT<br>R: AAGCATCATCGAAACGCTCTC
HK2	F: GAGCCACCACTCACCCTACT<br>R: CCAGGCATTCGGCAATGTG
LDHA	F: ATGGCAACTCTAAAGGATCAGC<br>R: CCAACCCCAACAACTGTAATCT
CDH1	F: CGACCCAACCCAAGAATCTA<br>R: AGGCTGTGCCTTCCTACAGA
CDH2	F: GACAATGCCCCTCAAGTGTT<br>R: CCATTAAGCCGAGTGATGGT
CK19	F: TTTGAGACGGAACAGGCTCT<br>R: AATCCACCTCCACACTGACC
SNAIL1	F: CACTATGCCGCGCTCTTTC<br>R: GGTCGTAGGGCTGCTGGAA
SNAIL2	F: GAGCATTTGCAGACAGGTCA<br>R: GCTTCGGAGTGAAGAAATGC
ABCB1	F: GCCTGGCAGCTGGAAGACAAATAC<br>R: ATGGCCAAAATCACAAGGGTTAGC
